# Diarylamidine activation of a brachiopod DEG/ENaC/ASIC channel

**DOI:** 10.1016/j.jbc.2024.108066

**Published:** 2024-12-10

**Authors:** Josep Martí-Solans, Aina Børve, Andreas Hejnol, Timothy Lynagh

**Affiliations:** 1 Michael Sars Centre, University of Bergen, Bergen, Norway; 2 Department of Biological Sciences, University of Bergen, Bergen, Norway

**Keywords:** ion channels, DEG/ENaC, ASIC, diarylamidine, diminazene, pentamidine

## Abstract

Diarylamidines are a group of widely used small molecule drugs. One common use of diarylamidines is their pharmacological inhibition of ligand-gated cation channels, including tetrameric ionotropic glutamate receptors and trimeric degenerin/epithelial sodium channel/acid-sensing ion channels. Here, we discover a degenerin/epithelial sodium channel/acid-sensing ion channel from the brachiopod (lamp shell) *Novocrania anomala*, at which diarylamidines act as agonists. The channel is closely related to bile acid–gated, pH-gated, and peptide-gated channels but is not activated by such stimuli. We describe activation of the channel by diminazene, 4′,6-diamidino-2-phenylindole, and pentamidine, examine several biophysical and pharmacological properties, and briefly explore the molecular determinants of channel activity with site-directed mutagenesis. We term this channel the diarylamidine-activated sodium channel.

Diarylamidines, such as pentamidine, diminazene, and 4′,6-diamidino-2-phenylindole (DAPI), are a class of chemical compounds distinguished by the presence of two aromatic amidine groups joined by a hydrophobic link. Diarylamidines are commonly utilized as antiparasitic drugs and cell nucleus dyes due to their capacity to strongly bind DNA AT-rich regions and interfere with DNA and RNA and thus protein synthesis (1). Interestingly, in addition to these capacities, diarylamidines are used as ion channels blockers. For example, various ionotropic glutamate receptors, which are mostly nonselective cation channels, are inhibited by diminazene, pentamidine, and/or DAPI (2, 3, 4). The inhibitory effect of diarylamidines is not restricted to ionotropic glutamate receptors, as several members of the degenerin/epithelial Na^+^ channel/acid-sensing ion channel (DEG/ENaC/ASIC) family of Na^+^ channels are also inhibited by diarylamidines (5, 6, 7, 8, 9, 10). Indeed, diminazene is emerging as a widely used DEG/ENaC/ASIC blocker because it may be more of a genuine pore blocker (*i.e.*, plugs the narrowest part of the pore) of more numerous DEG/ENaC/ASIC channels than amiloride, which has traditionally been used to characterize channel pharmacology (9).

The DEG/ENaC/ASIC family includes a diverse range of ion channels that play crucial roles in various physiological processes, including mechanotransduction, ion homeostasis, and synaptic transmission (11). Members of the DEG/ENaC/ASIC ion channel family have a conserved trimeric structure, with three homologous subunits arranged 3-fold symmetrically around a central pore (12). Each subunit has two transmembrane domains (TM1 and TM2) with a large extracellular region in between, and the N- and C-terminal domains are intracellular. TM1 and TM2 of each subunit form the channel pore, which is in most characterized cases moderately selective for Na^+^ over K^+^, usually with minimal divalent cation permeability (13). Because amino acid residue numbering differs greatly across various channels but TM2 sequence is relatively well conserved (14), comparison of TM2 residues in different channels is aided by numbering TM2 residues from 0′, a moderately conserved asparagine N0′, or aspartate D0′ near the top of the pore, down to, *e.g.*, highly conserved G6′ in the middle of the pore, and E18′ at the intracellular end of TM2 (15). Several amino acid residues in the mid to upper part of the pore, including 0′ and 6′ positions, appear to determine the binding of cationic channel blockers, including diminazene, the cationic diuretic amiloride, and Ca^2+^ ions (8, 9, 16).

Computational docking and mutagenesis in rat ASIC1a suggest that both diminazene and amiloride plug the channel pore between rings of glycine residues (G3′ and G6′) and aspartate residues (D0′) (9). An X-ray structure of chick ASIC1, in contrast, shows amiloride in two distinct sites: one amiloride molecule in each of the three “lateral fenestrations” between adjacent subunits at the upper/lateral entrance to the pore, close to D0’, and two stacked amiloride molecules in each of three more distal extracellular sites within single subunits, the “acidic pocket” (17). Cryo-electron microscopy structures of a more distantly related FMRFamide-gated DEG/ENaC/ASIC channel (FaNaC) in the presence of diminazene failed to resolve the drug, although a density was observed deep in the pore between TM2-G6′ residues, and mutagenesis of TM2 G6′ and to a lesser extent TM2-D0′ reduced potency of diminazene block (8). The upper half of TM1 and TM2 is intricately coupled to the extracellular domain *via* direct peptide link and also a membrane-vicinal loop from the extracellular domain, and this interface of membrane-spanning and extracellular domains is crucial both for channel activation by extracellular stimuli and for the activity of drugs that modulate channel function (17, 18, 19).

While investigating the molecular basis by which various stimuli activate channels in a particular branch of the DEG/ENaC/ASIC family, we tested the activity of several channels from invertebrate bilaterian animals, including *Novocrania anomala*, a brachiopod (lamp shell). To our surprise, one channel was activated, not inhibited, by diarylamidines. Here, we describe the biophysical and pharmacological properties of this unusual DEG/ENaC/ASIC channel.

## Results

### Diarylamidines gate a DEG/ENaC/ASIC channel from the brachiopod *N. anomala*

**Figure 1:**
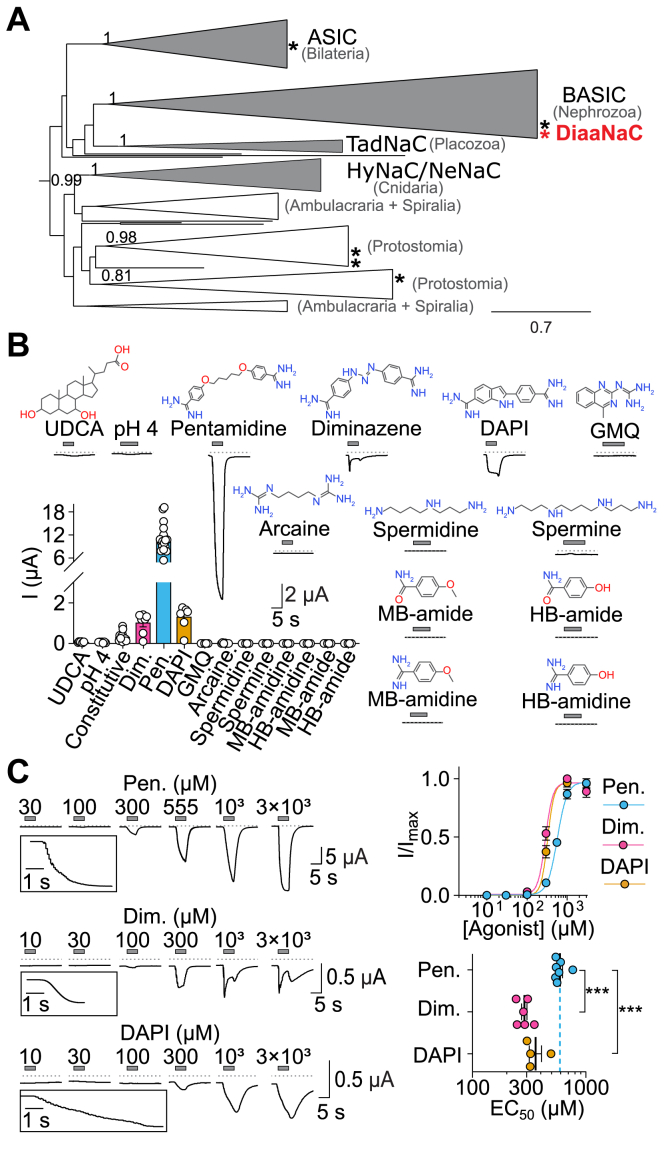
**DiaaNaC channel is activated by diarylamidines.***A*, ASIC/BASIC/HyNaC branch from clade A of the phylogenetic tree of the DEG/ENaC/ASIC family adapted from (20). Groups with functional data are shown in *gray* and groups without functional data in *white*. The taxonomic clades represented in each group are indicated in *gray*. *Asterisks* indicate *N. anomala* genes and *red asterisk* the gene characterized in this study (DiaaNaC). *B*, *lower left*, mean (column) and individual data point (n = 5–17 oocytes) current activated by potential stimuli. UDCA was tested at 2 mM, pentamidine at 300 μM, and all other compounds at 1 mM. *Above and right*, example current traces of *Xenopus* oocytes expressing DiaaNaC (*gray dotted line*: zero current level) and chemical structures (nonionized form) of tested compounds. *C*, *left*, example current traces from DiaaNaC activated by different diarylamidine concentrations in absence of Ca^2+^. *Upper right*, mean (±SEM) normalized current amplitude in response to increasing diarylamidine concentrations for DiaaNaC in the absence of Ca^2+^. *Insets*, magnified views of 3 mM (Pen. and DAPI)- or 300 μM (Dim.)-gated currents. *Lower right*, resulting EC_50_ values (individual data points on top of mean ± SEM) from n = 4 to 7 oocytes. ∗∗∗*p* < 0.001, one-way ANOVA. BASIC, bile acid-sensing ion channel; DAPI, 2-(4-carbamimidoylphenyl)-1H-indole-6-carboximidamide; DEG/ENaC/ASIC, degenerin/epithelial Na^+^ channel/acid-sensing ion channel; DiaaNaC, diarylamidine-activated Na^+^ channel; Dim., diminazene; GMQ, 2-(4-methylquinazolin-2-yl)guanidine; HB-amidine, 4-hydroxybenzamidine; HB-amide, 4-hydroxybenzamide; MB-amide, 4-methoxybenzamide; MB-amidine, 4-methoxybenzamidine; UDCA, ursodeoxycholic acid; Pen., pentamidine.

In a previous study (20), we performed a comprehensive phylogenetic analysis of the DEG/ENaC/ASIC family, identifying 11 DEG/ENaC/ASIC genes from the brachiopod (lamp shell) *N. anomala*. Notably, six of the *N. anomala* genes (asterisks in Fig. 1*A*) fall in the ASIC/bile acid-sensing ion channel (BASIC)/HyNaC branch within clade A DEG/ENaC/ASICs, one of two major clades that make up the family (20, 21, 22, 23). In the present study, we expressed a *N. anomala* gene that is closely related to mammalian BASICs (Fig. 1*A*, red asterisk; Nano_50439 in (20) and PP942623 in NCBI/GenBank) in *Xenopus laevis* oocytes and measured its activity with two-electrode voltage clamp. We sought to investigate if bile acids or protons, agonists of closely related BASIC and ASIC channels, would activate the *N. anomala* channel. Ursodeoxycholic acid and protons (pH 4), however, activated very little current—73.1 ± 10 nA and 64.6 ± 9 nA, respectively (Fig. 1*B*; n = 7). This suggests that, in contrast to closely related channels, the *N. anomala* channel does not significantly respond to these ligands.

Channels of the ASIC/BASIC/HyNaC branch show variable degrees of block by the antiparasitic agent diminazene, (5, 6, 10). Considering this, we investigated the effects of diminazene on the *N. anomala* channel, which in the absence of Ca^2^⁺ in the extracellular solution showed a modest constitutive current of 343 ± 70 nA (n = 12, Fig. 1*B*). We attempted to inhibit this current by applying diminazene at 1 mM, a concentration that completely blocks ASIC/BASIC/HyNaC channels (5, 6, 10). Unexpectedly, the application of diminazene activated substantial and reversible inward currents of 1.0 ± 0.2 μA (n = 7), without inhibiting the constitutive current (Fig. 1*B*). To investigate the specificity of this effect, we tested channel activation by other diarylamidines. Application of pentamidine (300 μM), which contains a flexible aliphatic chain, resulted in 12 times greater current amplitude than diminazene, whereas DAPI (1 mM) resulted in 1.3 times greater current amplitude than diminazene (Fig. 1*B*; n = 7–16). Therefore, we named this channel DiaaNaC (diarylamidine-activated Na^+^ channel).

We investigated requirements for agonist activity further, first by testing aliphatic polyamines arcaine, spermidine, and spermine, some of which modulate mammalian ASIC1, ASIC2, and/or ASIC3 (24, 25); however, neither activated DiaaNaC (Fig. 1*B*; n = 5–7), suggesting that the arylamidine moieties are important for agonist activity. We therefore tested the activity of monoarylamidines or monoarylamides, but none of these activated the channel (Fig. 1*B*; n = 5), indicating that the presence of two arylamidine groups is most probably necessary for channel activation.

We determined the potency of the different diarylamidines at DiaaNaC by applying the drugs in increasing concentrations. Diminazene and DAPI (EC_50_ 287.1 ± 16.5 and 361.6.1 ± 44.2 μM, respectively) showed higher potency than pentamidine (591.6 ± 30.7 μM, both *p* < 0.001 one-way ANOVA), despite much greater efficacy of the latter (Fig. 1, *B* and *C*). As solution exchange around large *X. laevis* oocytes can take up to a second, we could not measure the speed of channel activation; however, this appeared much slower with DAPI compared to pentamidine and diminazene (Fig. 1*C*, inset). We also found that high concentrations of diminazene blocked the diminazene-activated current (Fig. 1*C*), which we explore in detail later. Because of the greater amplitude and more complete deactivation of pentamidine-gated currents, we mostly used pentamidine in further studying channel properties.

### DiaaNaC is inhibited by amiloride and Ca^2^⁺

**Figure 2:**
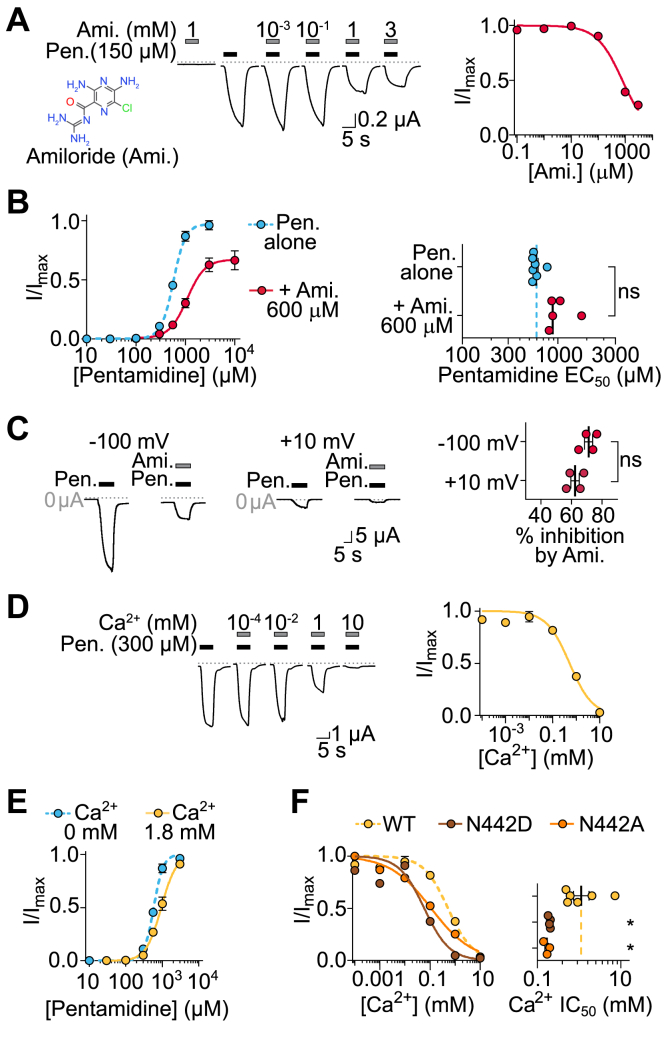
**Amiloride and Ca**^**2+**^**ions block DiaaNaC.***A*, *left-to-right*, amiloride structure (nonionized form), example pentamidine (150 μM)-gated currents in increasing amiloride concentrations, and mean (±SEM, n = 4) normalized current amplitude in increasing amiloride concentrations. *B*, *left*, mean (±SEM, n = 4) normalized pentamidine-gated current amplitude alone or with 600 μM amiloride. *Right*, EC_50_ values from 5 to 8 oocytes (points) mean shown as stick. *C*, *left*, example recordings and *right*, mean (±SEM, n = 4 oocytes) pentamidine (300 μM)-gated currents alone or with 1 mM amiloride at −100mV or +10mV membrane potential. *D*, *left*, example recording and *right*, mean normalized pentamidine (300 μM)-gated currents in increasing Ca^2+^ concentrations. *E*, mean (±SEM, n = 4) normalized pentamidine-gated current amplitude without Ca^2+^ or with 1.8 mM Ca^2+^. *F*, *left*, mean (±SEM, n = 4) normalized pentamidine (EC_10_ concentration)-gated current amplitude in response to increasing Ca^2+^ concentrations for indicated DiaaNaC mutants. *Right*, mean ± SEM (sticks) IC_50_ values for Ca^2+^ inhibition of WT and mutant channels at 3 to 6 oocytes (points). ∗*p* < 0.05 compared to WT, one-way ANOVA with Dunnett’s multiple comparisons test. Ami., amiloride; DiaaNaC, diarylamidine-activated Na^+^ channel; *ns*, not significantly different; Pen., pentamidine.

Diminazene and amiloride compete for a similar binding site in the pore of ASIC and BASIC channels (9, 10). Therefore, we investigated if amiloride could activate DiaaNaC-like diarylamidines, but the application of 1 mM amiloride, which strongly inhibits ASICs and BASICs, activated no current in DiaaNaC-expressing oocytes (Fig. 2*A*). Instead, when co-applied with pentamidine, amiloride inhibited pentamidine-gated currents, with an approximate IC_50_ of 800 ± 89 μM; n = 5, as we did not reach saturation of the inhibitory effect (Fig. 2*A*). In further experiments, pentamidine-gated currents were measured in the presence of 600 μM amiloride (Fig. 2*B*). The EC_50_ for pentamidine was not significantly altered, and higher concentrations of pentamidine did not relieve the inhibition by amiloride (Fig. 2*B*). This indicates that amiloride inhibition is not competitive (26), suggesting that amiloride inhibits *via* a different site than pentamidine activates. We questioned if amiloride inhibits *via* a site in the DiaaNaC channel pore and therefore tested inhibition at a depolarized membrane potential, which should weaken binding of such a positively charged molecule in the channel pore. However, the percentage of amiloride block did not change significantly when membrane voltage was changed from −100 mV to +10 mV (Fig. 2*C*). This suggests that amiloride block in DiaaNaC is not strongly voltage dependent and not *via* a site that is deep in the electric field across the membrane.

Next, we tested for potential inhibition of DiaaNaC by Ca^2+^, as inhibition by this divalent cation is broadly conserved in the ASIC/BASIC/HyNaC branch (5, 7, 27, 28). After measuring the amplitude of pentamidine-gated currents of DiaaNaC at various Ca^2+^ concentrations, we found an apparent IC_50_ of 0.5 ± 0.1 mM (Fig. 2*D*; n = 6) for Ca^2+^. This suggests that at likely physiological concentrations of extracellular Ca^2+^ [*e.g.*, ∼1 mM in rat and ∼10 mM in squid (29, 30)], DiaaNaC is largely inhibited. To investigate the antagonistic relationship of pentamidine and Ca^2+^, we measured the pentamidine concentration–response relationship at 1.8 mM Ca^2+^ and compared it to that in the absence of Ca^2+^. Ca^2+^ slightly shifted the concentration–response curve to the right (pentamidine EC_50_ with Ca^2+^ = 1.0 ± 0.1 mM; n = 6), and high concentrations of pentamidine could overcome the inhibitory effect of Ca^2+^, which we tentatively interpret as pentamidine and Ca^2+^ competing for overlapping binding sites. The negatively charged TM2 D0′ residue in the upper half of the rat ASIC1a pore is crucial for diminazene and Ca^2+^ activity (9, 16), and the homologous position in DiaaNaC is instead a neutral but isosteric asparagine residue, N0′ (N442). When we mutated DiaaNaC N0′ to aspartate or to small, nonpolar alanine, the IC_50_ for Ca^2+^ shifted to the left in both cases (IC_50; N442D_ = 0.06 ± 0.02 mM and IC_50; N442A_ = 0.1 ± 0.02 mM; Fig. 2*F*). Our simple nonlinear regression fit the N0′D mutant relatively poorly, possibly indicating more complex effects of Ca^2+^ on this mutant, although we did not explore this further. The fact that both alanine and aspartate substitution of the N0′ residue led to increased Ca^2+^ potency would suggest that Ca^2+^ inhibition of DiaaNaC does not involve binding to the N0′ side chain.

### Channel properties of DiaaNaC

**Figure 3:**
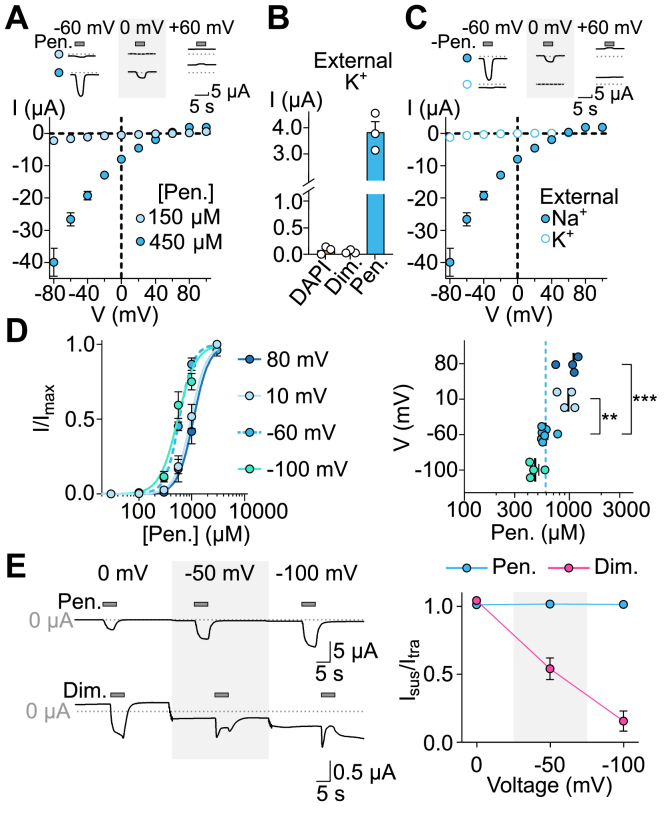
**DiaaNaC channel properties.***A*, *upper*, example recording (*gray dotted line*: zero current level), and *lower*, mean (±SEM, n = 5) pentamidine (150 and 450 μM)-gated current at different membrane potentials (n = 5). *B*, mean (±SEM, n = 3–5) DAPI (1 mM)-, diminazene (1 mM)-, and pentamidine (300 μM)-gated current at membrane potential −60 mV in the presence of external solution containing 140 KCl instead of NaCl. *C*, mean (±SEM, n = 5) pentamidine (450 μM)-gated current at different membrane potentials in extracellular NaCl or KCl (n = 5). *D*, *left*, mean (±SEM, n = 4) normalized pentamidine-gated current amplitude in response to increasing concentrations of pentamidine at different membrane potentials. *Right*, mean (±SEM, sticks; from n = 4–7 different oocytes, points) pentamidine EC_50_ compared by one-way ANOVA with Dunnett’s multiple comparisons test: ∗∗*p* < 0.01; ∗∗∗*p* < 0.001. *E*, *left*, example 1 mM pentamidine- (upper) or diminazene (lower)-gated currents at indicated membrane potentials. *Right*, mean (±SEM, n = 5) sustained current (I_sus_) amplitude normalized to transient current (I_trans_) amplitude at different membrane potentials. DAPI, 4′,6-diamidino-2-phenylindole; DiaaNaC, diarylamidine-activated Na^+^ channel; Dim., diminazene; Pen., pentamidine.

We investigated if pentamidine itself permeates the DiaaNaC channel, which could contribute to the inward currents in high extracellular concentrations of pentamidine, a divalent cation at physiological pH due to its two amidine groups (31). If pentamidine permeates DiaaNaC, the membrane potential at which the current reverses direction, or reversal potential (E_rev_), should shift toward more positive potentials in increased extracellular pentamidine concentrations. We tested this by measuring current–voltage relationships at two different concentrations of pentamidine at membrane potentials from −80 mV to 100 mV (Fig. 3*A*). We chose 150 and 450 μM because (a) a 3-fold increase in pentamidine concentration, if permeant, would substantially shift the reversal potential and (b) unlike higher concentrations of pentamidine, these concentrations lead to “moderate” current amplitudes that are not excessive, without having to titer the amount of DiaaNaC RNA injected. We saw no significant shift in E_rev_ with increasing pentamidine concentration (E_rev,150μM_ = 53.5 ± 5.0 mV and E_rev, 450μM_ = 57.6 ± 3.4 mV; n = 5). This was more difficult to assess for diminazene and DAPI, due to their smaller current amplitude; however, in the absence of extracellular Na^+^ and presence of 1 mM extracellular diminazene or DAPI, inward currents were tiny, if any, at −60 mV (Fig. 3*B*). We conclude that diarylamidines permeate DiaaNaC either not at all or much more slowly than Na^+^, although more hyperpolarized test potentials could be more revealing.

We also considered permeation of different monovalent cations through DiaaNaC. With typical high-Na^+^ extracellular solutions (in the absence of Ca^2+^), we measured a positive E_rev_ of 57.6 ± 3.4 mV (n = 5), suggesting that the currents through this channel are predominantly carried by Na^+^. When K^+^ was the only monovalent cation in our extracellular solution, pentamidine-gated currents were relatively small (Fig. 3*B*). This made it difficult to measure E_rev_ in this solution, but we estimated that E_rev_ = −12.2 ± 4.6 mV (n = 5) (Fig. 3*C*), a substantial shift in E_rev_. These data indicate that DiaaNaC strongly prefers Na^+^ to K^+^, in terms of both permeability and conductance.

We next evaluated the effect of membrane potential on the apparent affinity of pentamidine for DiaaNaC. If the pentamidine-binding site is in in the transmembrane region, as is the case for diarylamidines in other DEG/ENaC/ASIC channels (8, 9), changes in membrane potential should affect the affinity of the positively charged drug for DiaaNaC. Therefore, we assessed *apparent* affinity *via* concentration–response experiments at four different voltages (Fig. 3*D*). When membrane potential was positive (*i.e.*, +10 or +80 mV), the affinity of pentamidine for DiaaNaC decreased (EC_50, +10mV_ = 959.0 ± 80.7 μM and EC_50, +80mV_ = 1034 ± 101.2 μM; n = 4) compared to reference (EC_50, −60mV_ = 591.6 ± 30.7 μM; n = 7). Consistent with this, apparent affinity of pentamidine for DiaaNaC tended to increase at a more negative membrane potential (EC_50, −100mV_ = 471.0 ± 38.4 μM). These results show that pentamidine activity is weakly voltage dependent. This weak but significant effect of membrane potential on pentamidine potency could derive from pentamidine binding just within the electric field, *e.g.*, near the top of the membrane-spanning channel, or from membrane electric potential affecting the machinery of pentamidine-induced channel gating.

Unlike pentamidine and DAPI, diminazene-gated currents rapidly decreased in amplitude during application of the drug (Fig. 3*E*), especially at high concentrations (Fig. 1, *B* and *C*). We hypothesized that this may be from diminazene activating the channel *via* one site and blocking the channel pore *via* another. Consistent with this hypothesis, diminazene blocked close to 100% of the current at −100 mV, and block was progressively relieved with decreased negative potential (Fig. 3*E*). In contrast, we observed no such block by pentamidine, even at −100 mV.

### Molecular determinants of pentamidine activity

**Figure 4:**
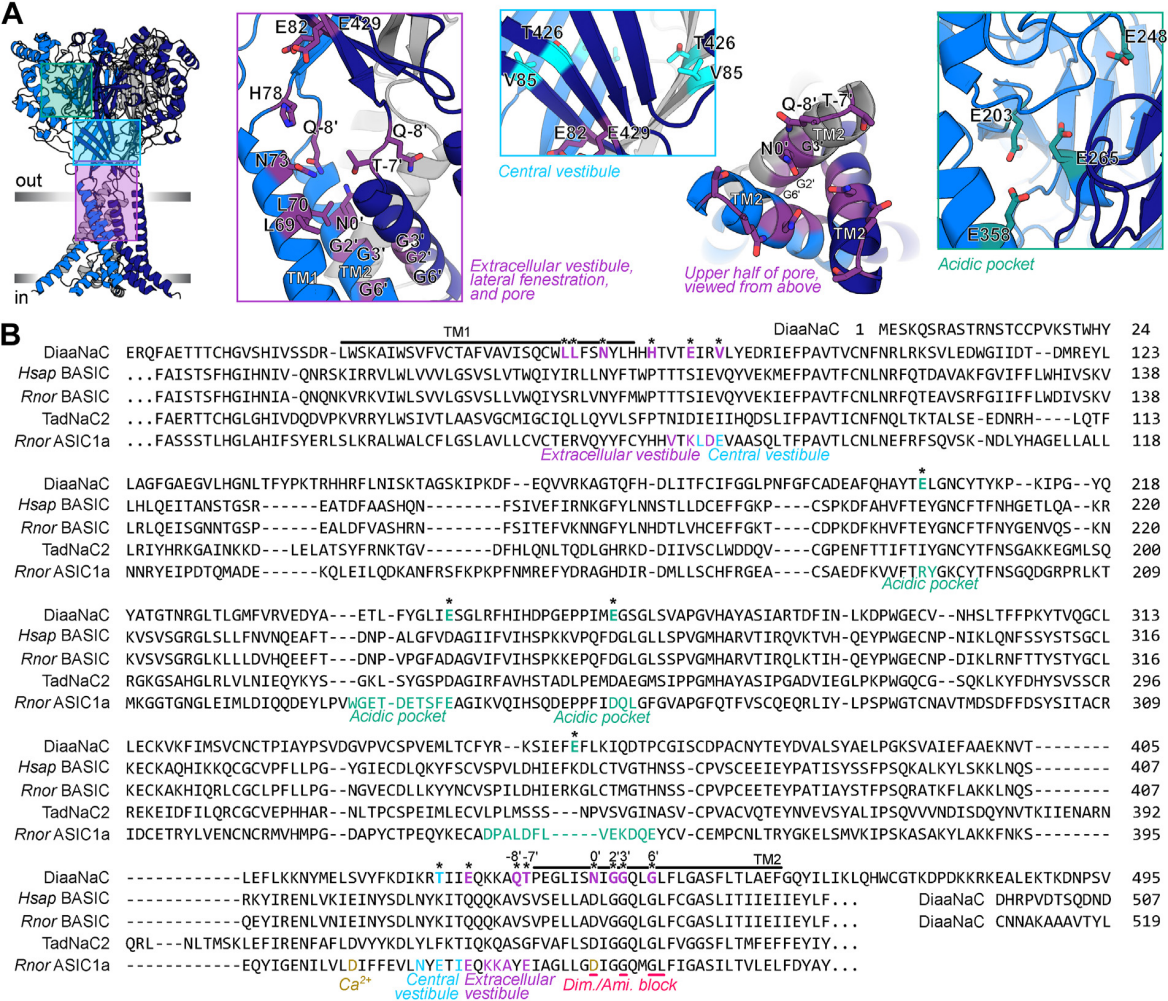
**DiaaNaC amino acid sequence and candidate determinants of function.***A*, AlphaFold structural model of DiaaNaC (*left*), with selected regions magnified and amino acid residues labeled (*middle to right*). TM1 and TM2, transmembrane helices 1 and 2 within each subunit. *B*, amino acid sequence alignment of DiaaNaC (full sequence) with selected closely related DEG/ENaC channels (excluding N and C termini) from *gray* branches in Fig. 1*A*. *Asterisks* indicate DiaaNaC residues mutated here, color-matched to (*A*). *Colored* and labeled residues in other channel sequences are implicated in indicated functions in previous studies on those channels. TM2 0′ is a way of referring to homologous residues from numerous DEG/ENaC channels that would otherwise have differing residue numbers. Ami., amiloride; DiaaNaC, diarylamidine-activated Na^+^ channel; Dim., diminazene; *Hsap*, homo sapiens; *Rnor*, rattus norvegicus.

Using site-directed mutagenesis, we made some attempt to identify amino acid residues that determine activation of DiaaNaC by diarylamidines. This search was not exhaustive, instead we focused on selected putative structural regions of the channel, as inferred from our AlphaFold 3D model (32) of DiaaNaC (Fig. 4*A*). First, the top half of the channel pore, which is narrow near the middle of the bilayer but opens into an “extracellular vestibule” and “lateral fenestrations” at the outer surface of the bilayer (purple in Fig. 4*A*), because pentamidine potency was weakly voltage dependent, the pore has been implicated in diminazene block of ASICs and FaNaCs (8, 9), and the lateral fenestrations provide a potential drug pathway (17). Second, the “central vestibule” (cyan in Fig. 4*A*), because certain small-molecule agonists or peptidic modulators of ASICs are thought to act *via* this site (33, 34, 35). Finally the “acidic pocket”, as this pocket seems dynamic during activation of diverse Deg/ENaC/ASICs (8, 36), it was proposed as a diarylamidine-binding site in ASICs in computational dockings (6) and contains several acidic residues in DiaaNaC that could foreseeably interact with positively charged diarylamidines (teal in Fig. 4*A*). We made a total of 19 DiaaNaC mutants, each containing a single amino acid substitution in one of these putative regions, and tested responses to pentamidine in oocytes injected with mutant RNAs.

**Figure 5:**
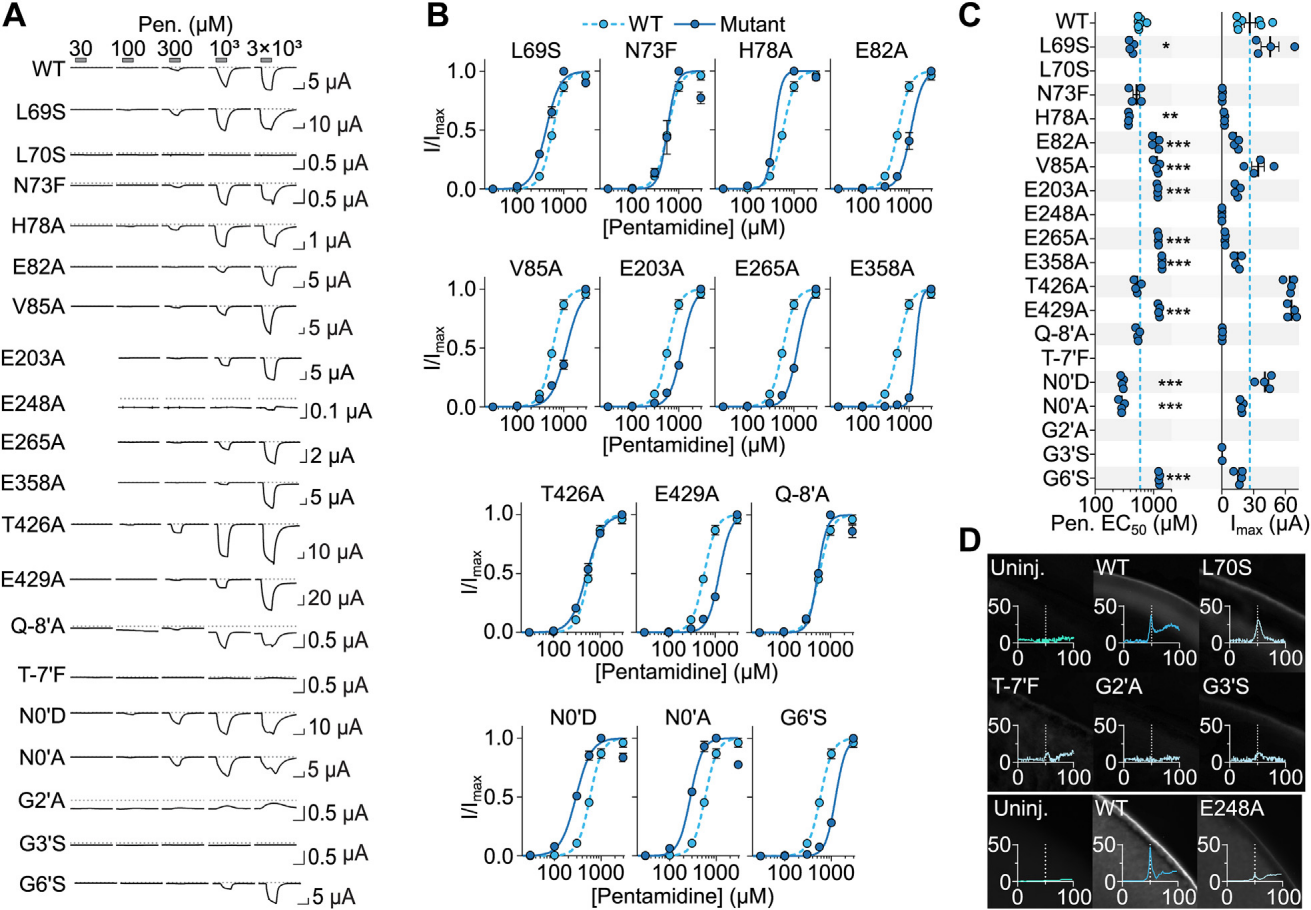
**Pentamidine potency at DiaaNaC mutants.***A*, example current responses to pentamidine (Pen.) at oocytes expressing indicated DiaaNaC constructs. *x*-axis scale bars: 5 s. *B*, mean (±SEM, n = 4) normalized current amplitude in response to increasing pentamidine concentrations for DiaaNaC mutants. C, *left*, mean [*sticks* (not always visible) ± SEM, n = 4] and data points for pentamidine EC_50_ of indicated mutants. ∗*p* < 0.05; ∗∗*p* < 0.01; ∗∗∗*p* < 0.001 different to WT in one-way ANOVA with Dunnett’s multiple comparisons test. *Right*, same but maximum current amplitude (I_max_). *D*, immunolabeling of c-Myc tag in DiaaNaC mutants. Micrographs shown, and y, *gray value*, is plotted against *x*, distance from out-to-in (μM); *white dashed line* indicates presumptive cell membrane. E248A from a second batch of experiments, hence additional control panels. DiaaNaC, diarylamidine-activated Na^+^ channel.

T-7′F (lining the lateral fenestration) and G2′A and G3′S (deep in the channel pore) mutations drastically reduced oocyte surface expression, evident in the absence of both pentamidine-gated currents and surface immunolabeling in oocytes expressing these mutants (Fig. 5, *A–D*). N0′D, N0′A, and G6′S mutant channels, in contrast, showed large pentamidine-gated currents akin to WT channels (Fig. 5, *A–C*). However, compared to WT channels (pentamidine EC_50_ 591.6 ± 30.7 μM), N0′D and N0′A mutations caused a significant 2-fold increase in pentamidine potency, whereas the G6′S mutation caused a significant 2-fold decrease in pentamidine potency (Fig. 5, *A–C*).

L69S, L70S, and N73F (in TM1); H78A, E82A, and E429A (in extracellular β-sheets); and Q-8′A (at the top of TM2) mutations were also designed to probe the role of residues lining the lateral fenestrations and extracellular vestibule (Fig. 4*A*). The L70S mutation completely abolished pentamidine-gated currents, despite apparent oocyte surface expression of L70S channels (Fig. 5, *A*, *C* and *D*). The other mutants of this region were more similar to WT, although L69S and H78A, both on the “left” of the lateral fenestration, caused a small but significant increase, and E82A and E429A, both at the “top” of the lateral fenestration and extracellular vestibule, caused a small but significant decrease in pentamidine potency (Fig. 5, *A–C*). V85 and T426 side chains orient into the *central* vestibule, just “above” the extracellular vestibule (Fig. 4*A*). The V85A mutation caused a small but significant decrease in pentamidine potency, whereas T426A mutant channels responded to pentamidine much like WT channels (Fig. 5, *A–C*).

The final region we assayed was the putative acidic pocket, where several glutamate side chains are in close proximity, including E203, E248, E265, and E358 (Fig. 4*A*). Alanine substitution at each of these positions was noticeably detrimental to function (Fig. 5, *A–C*), although in the case of E248A channels, this may be due to reduction in oocyte surface expression (Fig. 5*D*). E203A, E265A, and E358A all decreased pentamidine potency, with significant ∼2-fold increases in pentamidine EC_50_ values compared to WT (Fig. 5, *A–C*). Together, these results show that amino acid residues in very distinct parts of DiaaNaC contribute to pentamidine potency, from deep in the pore, through the vestibules and fenestrations, up to the acidic pocket, although the L70S mutation in the lateral fenestration had the most detrimental effect on pentamidine-gated currents.

### Molecular determinants of diminazene activity

From the above results, we cannot pinpoint a diarylamidine-binding site. However, activation by pentamidine was only weakly voltage dependent, whereas channel block by diminazene was strongly voltage dependent (Fig. 3), tentatively suggesting that diminizene blocks by a site deep in the pore, and pentamidine activates by a different site. To explore this further, and to test if activation by diminazene and pentamidine relies on the same amino acid residues, we performed several experiments with diminazene.

**Figure 6:**
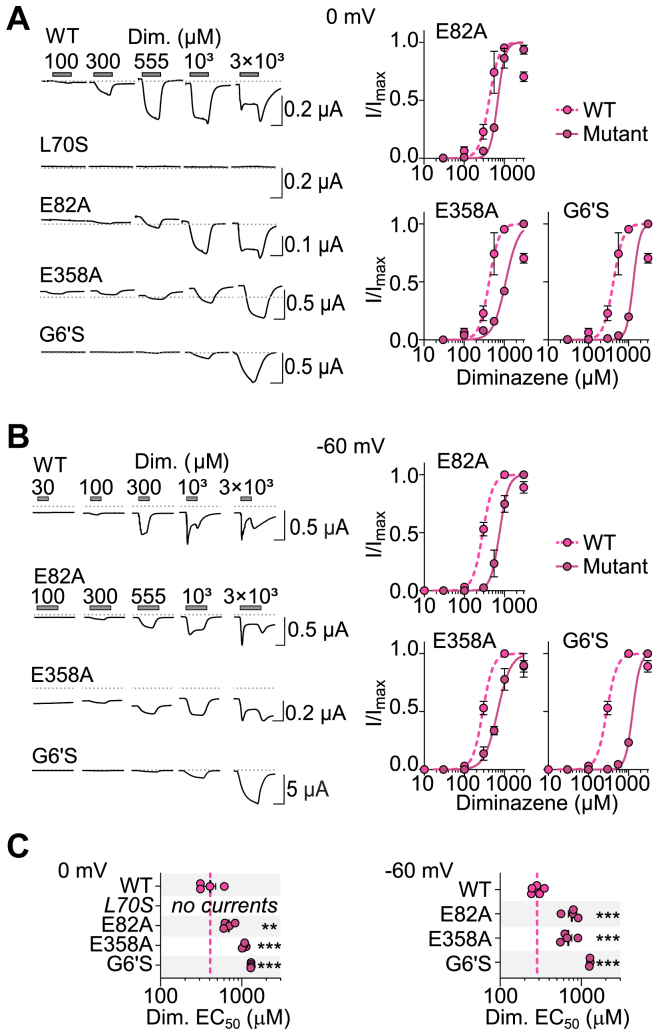
**Diminazene potency at selected DiaaNaC mutants.***A* and *B*, *left*, example current responses to diminazene (Dim.), and *right*, mean (±SEM, n = 3–4) normalized current amplitude in response to increasing Dim. Concentrations at oocytes expressing indicated DiaaNaC constructs, at a membrane potential of 0 mV (*A*) or −60 mV (*B*). WT recording in (*B*) is repeated from Fig.1*C*. *C*, mean [*sticks* (not always visible) ± SEM, n = 3 to 4] and data points for Dim. EC_50_ of indicated mutants. DiaaNaC, diarylamidine-activated Na^+^ channel.

First, we sought to compare diminazene potency between WT channels and four mutants that showed reduced pentamidine potency—G6′S from deep in the pore, L70S from the lateral fenestration, E82A from the top of the extracellular vestibule, and E358A from the acidic pocket. We sought to do this in the absence of channel block by measuring responses to increasing diminazene concentrations at a holding potential of 0 mV (Fig. 6*A*). This yielded a diminazene EC_50_ for activation of 409 ± 71 μM at WT channels, significantly higher (less potent) than WT at −60 mV (287 ± 17 μM, Fig. 6, *A–C*). Whether this difference is due to diminazene block curtailing the responses to high concentrations at −60 mV and shifting the EC_50_ to lower concentrations (compare WT currents in Fig. 6, *A* and *B*) or due to the negative potential increasing binding affinity, we do not know. Nonetheless, at 0 mV, block was absent at most diminazene concentrations (Fig. 6*A*), and compared to WT, diminazene-gated currents were abolished at L70S channels, and potency was significantly reduced at E82A, E358A, and G6′S channels (Fig. 6, *A* and *C*). This is remarkably similar to the effects of these mutations on pentamidine potency (Fig. 5, *A–C*), offering evidence that activation by diminazene and pentamidine is determined by the same amino acid residues in DiaaNaC.

Second, we tested mutant channel responses to diminazene at −60 mV. The goal of these experiments was to determine if mutations that reduced potency of activation by diminazene also reduced channel block by diminazene. At −60 mV, again each mutation caused a decrease in agonist potency compared to WT (Fig. 6, *B* and *C*); however, channel block was affected differently by the mutations. At oocytes expressing WT channels, block can be seen to develop arguably during the application of 300 μM and certainly during the application of 1 mM and 3 mM diminazene (Fig. 6*B*, left). In channels with the E82A mutation in the extracellular vestibule, few channels are activated by 300 μM diminazene, and noticeable block develops with 1 and 3 mM diminazene. Perhaps not surprisingly, in channels with the G6′S mutation in the channel pore, block was not evident, even at 3 mM diminazene (Fig. 6*B*, left). More remarkably, in E358A channels, where an acidic side chain far from the channel pore is neutralized, block occurs only at 3 mM diminazene, much higher than at WT (Fig. 6*B*, left).

In summarizing, mutations that decrease pentamidine potency also decrease diminazene potency. Although a mutation deep in the DiaaNaC pore is the only mutation that *abolishes* channel block by diminazene, among the other mutations that decrease agonist potency of diminazene, some of them also seem to decrease channel block.

## Discussion

DiaaNaC shares several biophysical properties with many other DEG/ENaC/ASIC channels, including greater Na^+^ than K^+^ permeation and inhibition by Ca^2+^ and amiloride, as expected from primary sequence homology, especially in the channel domain. While closely related ASICs are inhibited by the diarylamidines diminazene, pentamidine, DAPI, and hydroxystilbamidine, and BASICs, HyNaCs, “TadNac” (a closely related placozoan channel), and even more distantly related FaNaCs, are inhibited by diminazene (5, 6, 7, 8, 9, 10), DiaaNaC is *activated* by diminazene, pentamidine, and DAPI in the high micromolar to low millimolar range.

Notably, while diminazene from 300 μM upward activates DiaaNaC, diminazene at 1 mM and higher also blocks DiaaNaC, unlike pentamidine and DAPI. This block is abolished by mutating the highly conserved G6′ residue deep in the channel pore, as is the case in FaNaCs (8), and mutations further from the membrane did not abolish diminazene block. This, combined with strong voltage dependence of diminazene block and the low potency of diminazene block of channels with a neutral N0′ residue in the top of the pore relative to those with a negatively charged D0′ residue, suggests that diminazene block is *via* a binding site deep in the pore, whereby at least one of the positively charged amidines interacts with TM2 0′ side chains (6, 8, 9). Although not explored at length here, amiloride block is different, in that it is not voltage dependent in DiaaNaC, in contrast to amiloride block of rat ASIC1a (9).

Regarding agonist activity, we cannot pinpoint a binding site, but we learn that (1) mutations in several distinct parts of the channel can increase or decrease pentamidine potency, (2) mutations that decrease pentamidine potency also decrease diminazene agonist potency, and (3) the agonist effects of pentamidine and diminazene are significantly but only weakly sensitive to membrane potential. We therefore think that diarylamidine agonists share an agonist binding site that is distinct from the pore blocking site of diminazene; however, the two sites are curiously linked: although extracellular vestibule and acidic pocket mutations that reduced agonist potency did not abolish channel block, they did appear to reduce potency of the block. This link could be merely indirect if channel block by diminazene is slow, as is the case in ASICs (9), in which case mutant DiaaNaC channel open probability is too low for slow entry of diminazene at concentrations otherwise capable of blocking the open DiaaNaC channel. Alternatively, the two sites may be overlapping, such that certain mutations can easily affect both agonist and blocking activity.

The latter might be achieved by an agonist site in the putative lateral fenestration of DiaaNaC, which dips into the outer leaflet of the membrane. Indeed, the largest decrease in potency—a total loss of agonist activity—among the mutants tested, L70S, was at this location. With predicted water/octanol partition coefficients of around two (37), diarylamidine agonists could perhaps open the channel by associating with both the lipid bilayer and the upper half of the channel. Certainly, several modulators of ASICs seem to act *via* this region, as evidenced by structural data capturing amiloride in the lateral fenestration and the large toxin agonist MitTx binding close by (17) and by mutations here that decrease allosteric inhibition by ibuprofen (19).

Other possible agonist binding sites could be more distal to the membrane, including the central vestibule [also called the “nonproton ligand-sensing domain” in ASICs (35)] or the even more distal acidic pocket, as we find that mutations in both sites, especially the latter, decrease agonist potency of pentamidine and diminazene. Although this possibility seems inconsistent with the weak but significant voltage dependence of pentamidine and diminazene activation, voltage dependence of extracellular agonists can arise from an effect of membrane potential on the gating machinery (38). Furthermore, the central vestibule and the acidic pocket have been implicated in agonist or modulator binding in several DEG/ENaC/ASIC channels. Diminazene itself, together with a derivative of higher inhibitory potency, were computationally predicted to bind to the acidic pocket of ASICs (6, 39), although experimental studies in ASICs support only a channel pore binding site (9, 40). If diarylamidines do not activate DiaaNaC *via* the acidic pocket, the consistent decreases in agonist potency caused by mutations here would presumably be due to the mutations hindering conformational changes that are simply part of the gating process. Consistent with this notion, acidic pocket closure seems to be a conserved dynamic feature of channel activation, evidenced so far in ASICs, upon protons binding to numerous sites (36, 41), and in FaNaCs, upon activation by FMRFamide binding to an even more distal site (8, 42). Moreover, in the close relative of DiaaNaC, rat BASIC, an A387S mutation in the extracellular domain alters not only Ca^2+^ and amiloride blocking potency but also ion permeability (28).

Regarding the central vestibule, amiloride and several other cationic drugs, including arcaine, GMQ, and spermine, can activate or modulate certain ASICs *via* this site (24, 25, 43). However, none of these compounds activated DiaaNaC. Curiously, there is mutagenesis, modeling, and/or structural evidence for amiloride binding sites in the channel pore, in the lateral fenestrations, in the central vestibule, and/or in the acidic pocket of various ASICs, raising the possibility that the complex effects of many of these drugs may be *via* binding to multiple sites, as recently discussed for amiloride (44).

The biological role of DiaaNaC in *N. anomala* is even more mysterious. We are unaware of naturally occurring diarylamidines, and none of the related naturally occurring compounds we tested, such as arcaine or polyamines, activated the channel. We therefore presume there is a native agonist that remains to be identified. DiaaNaC is strongly inhibited by low millimolar concentrations of Ca^2+^ ions, and Ca^2+^ concentrations not only in seawater but also in extracellular fluid of squid can reach ∼10 mM (29) (we could not find such literature on brachiopods). It is thus foreseeable that native channel activity in the brachiopod might differ from what we see upon heterologous expression and typical laboratory settings. Curiously, we see no ortholog of DiaaNaC in transcriptomes of other brachiopods we examined, including *Terebretalia transversa* and *Lingula anatina*. If agonist activity of diarylamidines is indeed unique for *N. anomala* DiaaNaC, diminazene remains a good tool for inhibiting DEG/ENaC/ASICs in most native preparations.

## Experimental procedures

### Identification of *N. anomala* DiaaNaC, molecular biology, and chemicals

Transcript Nano_50439, renamed DiaaNaC here, was a *N. anomala* transcript identified as a DEG/ENaC/ASIC gene in a recent phylogenetic analysis of ours (20). The DiaaNaC full-length coding sequence was amplified by PCR from *N. anomala* cDNA and subcloned *via* SalI and BamHI sites into a pSP64 (polyA) vector (P1241, Promega) modified to contain the *X. laevis* globin 5′ and 3′ UTRs and a C-terminal Myc-tag linked to the final DiaaNaC residue *via* Gly-Ser linker (the BamHI site) (see (20) for full vector sequence). The cDNA sequence was deposited in NCBI GenBank accession number PP942623. Recombinant PCR with Phusion High-Fidelity DNA polymerase (F-549L, Thermo Fisher Scientific) was used to perform site-directed mutagenesis, as described previously (45). Sanger sequencing of the whole insert (Genewiz) corroborated the sequences of WT and mutant constructs. EcoRI (FD0274, Thermo Fisher Scientific) was used to linearize the cDNAs *via* a site shortly after the poly(A) sequence, and the mMESSAGE mMACHINE SP6 Transcription Kit (AM1340, Thermo Fisher Scientific) was used to synthesize cRNA.

Diminazene aceturate (D7770), pentamidine isethionate (P0547), spermidine trihydrochloride (S2501), and standard chemicals were purchased from Merck. DAPI (D1306) and other standard chemicals were purchased from Fisher Scientific. Other chemicals were purchased from other companies: 4-methoxybenzamidine hydrochloride (F300402), 4-hydroxybenzamidine hydrochloride (F023564), 4-hydroxybenzamide (F002826), 4-methoxybenzamide (F240987) from Fluorochem; arcaine sulfate (A-285) from Alomone Labs; and sodium ursodeoxycholic acid (sc-222407) from Santa Cruz Biotechnology. The 2D chemical structures were drawn with PubChem Sketcher V2.4 (46).

### Heterologous expression and detection of surface expression

WT cRNA (12 ng) for most experiments with WT channels and 48 ng of WT (for additional diminazene experiments and for comparing pentamidine potency of mutants) and mutant cRNA were injected into stage V/VI *X. laevis* oocytes purchased from Ecocyte Bioscience. Before electrophysiological recordings or surface expression determination, the oocytes were cultured for 3 days at 18 °C in 50% Leibowitz medium (L1518, Merck), supplemented with 0.25 mg/ml gentamicin, 1 mM L-glutamine, and 15 mM Hepes (pH 7.6). For surface expression detection, mouse anti-Myc tag monoclonal IgG1 antibody (MA121316, Fisher Scientific) was used to detect the myc tag attached to the 3′-end of all the constructs used in this work, and the protocol previously used in our lab was followed (47) using a Zeiss Axio Scope A1 microscope. The intensity of the expression signal was quantified by tracing a perpendicular line across the oocyte membrane and measuring the gray values with ImageJ software (48).

### Electrophysiological recordings and data analysis

Whole cell currents were recorded from oocytes by two-electrode voltage clamp using an OC-725D amplifier (Warner Instruments) controlled *via* an Axon Digitata 1550B interface and pClamp v11 (Molecular Devices), acquired at 1 kHz, and filtered at 200 Hz. Currents were analyzed in pClamp v10.7 software (Molecular Devices) and additionally filtered at 10 Hz for display in figures. Oocytes were clamped at −60 mV, unless otherwise indicated, and continuously perfused with a bath solution containing (in mM): 140 NaCl, 1.8 BaCl_2_, and 10 Hepes. The pH was adjusted to 7.5 with NaOH, HCl, or KOH, as appropriate. For current–voltage relationships where K^+^ replaced Na^+^, KCl replaced NaCl in the above bath solution. In most experiments, activating solution was applied to oocytes in between resting periods of at least 30 to 60 s. After retrieving current amplitude from pClamp, all data were analyzed in Prism v9 (GraphPad Software). Currents were normalized by dividing by the maximum current at that oocyte, giving “I/I_max_” in most concentration–response graphs. For graphs in figures and for calculating half-maximal effective (or inhibitory) concentration (EC_50_ or IC_50_) values, variable slope nonlinear regression was used in Prism v9, with the equation Y = Bottom + (Top-Bottom)/ + 1 + 10^∧^((LogEC50-X)∗HillSlope)), and maximum was constrained to one and minimum to zero. Curves in figures are fit to the mean data points. EC_50_ and IC_50_ values reported in text and used in statistical comparisons are means from curve fits to data on individual oocytes.

### Structural model

The DiaaNaC trimeric structure was predicted using AlphaFold3 on the Google Colab site (32). The model was visualized in Fig. 4 with PyMol v2.4 (Schrödinger).

## Data availability

All data supporting the conclusions of this article are presented in the main manuscript. Additional raw data will be made available by the authors on request. DiaaNaC nucleotide sequence was deposited in NCBI/GenBank under PP942623.

## Conflict of interest

The authors declare that they have no conflicts of interest with the contents of this article.
